# AVRA: Automatic visual ratings of atrophy from MRI images using recurrent convolutional neural networks

**DOI:** 10.1016/j.nicl.2019.101872

**Published:** 2019-05-25

**Authors:** Gustav Mårtensson, Daniel Ferreira, Lena Cavallin, J-Sebastian Muehlboeck, Lars-Olof Wahlund, Chunliang Wang, Eric Westman, for the Alzheimer's Disease Neuroimaging Initiative

**Affiliations:** a Division of Clinical Geriatrics, Department of Neurobiology, Care Sciences and Society, Karolinska Institutet, Stockholm, Sweden; b Department of Clinical Neuroscience, Karolinska Institutet, Stockholm, Sweden; c Department of Radiology, Karolinska University Hospital, Stockholm, Sweden; d School of Technology and Health, KTH Royal Institute of Technology, Stockholm, Sweden; e Department of Neuroimaging, Centre for Neuroimaging Sciences, Institute of Psychiatry, Psychology and Neuroscience, King's College London, London, UK

**Keywords:** Atrophy, Visual ratings, Machine learning, MRI, Neuroimaging, Radiology

## Abstract

Quantifying the degree of atrophy is done clinically by neuroradiologists following established visual rating scales. For these assessments to be reliable the rater requires substantial training and experience, and even then the rating agreement between two radiologists is not perfect. We have developed a model we call *AVRA* (Automatic Visual Ratings of Atrophy) based on machine learning methods and trained on 2350 visual ratings made by an experienced neuroradiologist. It provides fast and automatic ratings for Scheltens' scale of medial temporal atrophy (MTA), the frontal subscale of Pasquier's Global Cortical Atrophy (GCA-F) scale, and Koedam's scale of Posterior Atrophy (PA). We demonstrate substantial inter-rater agreement between AVRA's and a neuroradiologist ratings with Cohen's weighted kappa values of *κ*_*w*_ = 0.74/0.72 (MTA left/right), *κ*_*w*_ = 0.62 (GCA-F) and *κ*_*w*_ = 0.74 (PA). We conclude that automatic visual ratings of atrophy can potentially have great scientific value, and aim to present AVRA as a freely available toolbox.

## Highlights

• A novel method for automatic visual ratings of atrophy from MRI images.
• Substantial inter-rater agreement to radiologist for MTA, frontal GCA and PA scales.
• Runs in <1 min/image and requires no prior preprocessing.

## 1 Introduction

The assessment of structural changes in the brain is made clinically by visual ratings of brain atrophy according to established visual rating scales. They offer an efficient and inexpensive method of quantifying the degree of atrophy and can help to improve the specificity and sensitivity of dementia diagnoses (Harper et al., 2015; Wahlund et al., 2017). However, there are limitations associated with visual ratings of atrophy, which may explain why they are still not widely used in the clinical routine. First, the ratings are inherently subjective which means that the agreement between two radiologist might be low if they have not had sufficient training (Harper et al., 2015). Second, in order to achieve adequate reliability the radiologist needs to be experienced and regularly perform ratings for the reproducibility not to drop (Cavallin et al., 2012a). Third, the ratings are relatively time consuming and tedious. It takes a few minutes per image (Wahlund et al., 1999), depending on rating scale and level of rating experience. While this amount of time may be feasible in most clinical settings, it does not easily allow studying large imaging cohorts of potentially thousands of images. An automatic method would remove the inter- and intra-rater variability and eliminate the time-consuming process of rating.

**Fig. 1:**
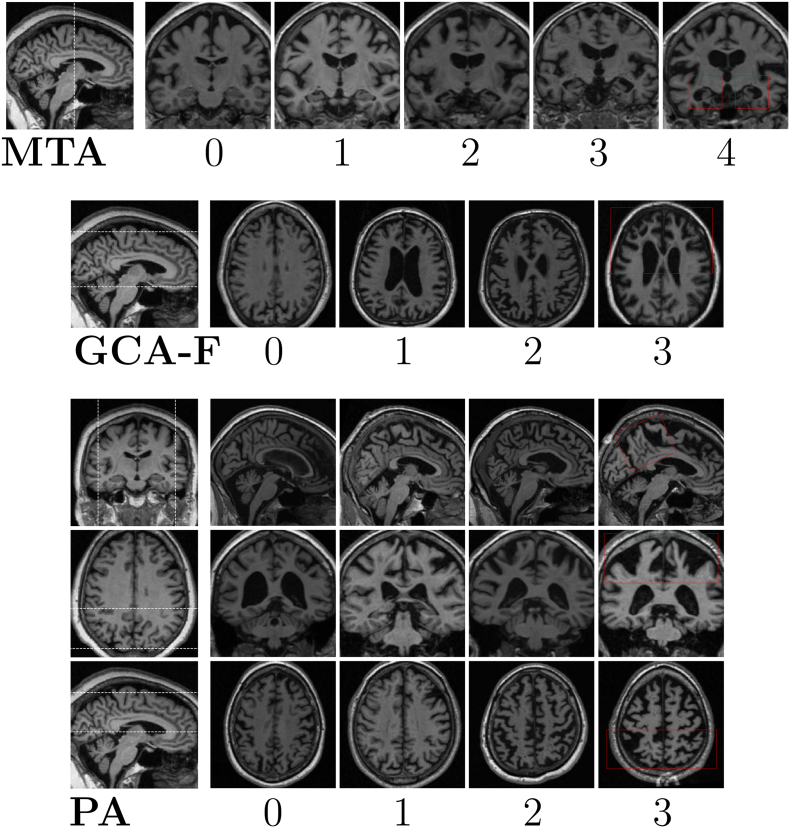
Examples of Scheltens' MTA scale (Scheltens et al., 1992), Pasquier's frontal subscale of GCA (Pasquier et al., 1996), and Koedam's PA scale (Koedam et al., 2011). The MTA ratings are done in the coronal plane, GCA-f in the axial plane, and PA ratings are based on assessments of all three planes. The area between the dashed lines in the left images indicates the slices assessed by a radiologist for the GCA-F and PA scales, while it shows the single slice assessed for MTA. The red boxes indicate the regions assessed for each rating scale.

Amongst the most commonly used visual rating scales—both in research and in clinical routine—are Scheltens' Medial Temporal Atrophy (MTA) scale (Scheltens et al., 1992), Koedam's scale for Posterior Atrophy (PA) (Koedam et al., 2011) and the frontal subscale of Global Cortical Atrophy (GCA-F) proposed by Pasquier (Scheltens et al., 1997; Pasquier et al., 1996). These scales each assess the atrophy in a specific region of the brain from Magnetic Resonance Imaging (MRI) or Computer Tomography (CT) images, and details about these scales can be seen in Table 1 with illustrative examples in Fig. 1. These scales have previously been validated by quantitative neuroimaging techniques used in research (Bresciani et al., 2005; Cavallin et al., 2012b; Wahlund et al., 1999; Möller et al., 2014; Ferreira et al., 2016). MTA ratings have been shown to be significantly (anti-)correlated with hippocampal volume and width of the temporal horn (Bresciani et al., 2005; Cavallin et al., 2012b; Wahlund et al., 1999). Möller et al. (2014) found statistical differences in parietal cortex between PA ratings both in volumes of specific posterior gray matter regions, and using voxel-based morphometry (Möller et al., 2014). Further, the GCA-F scale has been shown to reliably reflect atrophy in the frontal cortex using both volumetrics and surface-based analysis (Ferreira et al., 2016). Some studies have explicitly compared diagnostic ability of using regional cortical volume and thickness measures as opposed to visual ratings in dementia cohorts, showing improved discrimination when using cortical measures (Bresciani et al., 2005; Westman et al., 2019). However, in cases where a neuroimaging software fails to extract volumetric information (e.g. due to presence of image artifacts or an odd scanning protocol) it would not be feasible—nor possible—to manually correct this error in a clinical situation. Yet, it is likely that the radiologist would still be able to make visual atrophy ratings of those images.

**Table 1:** Description of Scheltens' MTA scale, Pasquier's frontal GCA subscale (GCA-F) and Koedam's PA scale. Abbreviations: Posterior cingulate sulcus (PCS); Parieto-occipital sulcus (POS); Precuneus (PRE); Hippocampus (HC).

Rating	MTA (Scheltens et al., 1992)	GCA-F (Pasquier et al., 1996)	PA (Koedam et al., 2011)
0	Normal	No atrophy	No atrophy
1	Widening of choroid fissure.	Mild sulcal atrophy in frontal lobe.	Mild widening of PCS and POS, mild atrophy of PRE and parietal lobes.
2	Increased widening of choroid fissure, widening of temporal horn, decreased height of HC.	Moderate sulcal atrophy in frontal lobe.	Substantial widening of PCS and POS, substantial atrophy of PRE and parietal lobes.
3	Increased widening of choroid fissure and temporal horn, further decreased heigth of HC.	Severe sulcal atrophy in frontal lobe.	Evident widening of PCS and POS, end-stage atrophy of PRE and parietal lobes.
4	Further decreased height of HC.	–	–
Rating slice(s)	Single coronal slice.	Multiple axial slices.	Multiple slices, all anatomical planes.

A few automatic (or semi-automatic) methods to quantify medial temporal atrophy—besides volumetrics—have previously been proposed. Two of them involve planimetrics based on manual delineation of hippocampus and surrounding structures that are combined into a single score of medial temporal atrophy (Zimny et al., 2013; Menéndez-González et al., 2014). While these methods assess almost the same structures as Scheltens' MTA scale, the different scales are not interchangeable and do not necessarily reflect the same atrophy patterns. Another study recently reported an automatic method that is trained on radiologist ratings which predicts MTA scores based on volumetric measures extracted from the MRI image (Lotjonen et al., 2017). Volumetric measures of brain regions can not be extracted from most CT images nor do they retain any information regarding the shape of the structures. It is reasonable to assume that the shapes are important since the visual MTA rating is done on a single slice, from which it is not possible to estimate the hippocampal volume.

Deep learning—a branch of machine learning—has recently generated impressive results in several fields, such as speech recognition, text semantics, image recognition and genomics (Lecun et al., 2015). Convolutional neural networks (CNN's) have already been substantially applied in medical image analysis (for recent reviews, see (Shen et al., 2017; Litjens et al., 2017)). In neuroimaging, deep neural networks have been used successfully for automatic methods of skull stripping (Roy et al., 2017; Kleesiek et al., 2016), brain age prediction (Cole et al., 2017), brain segmentation (Chen et al., 2018), PET image enhancement (Wang et al., 2018) and brain tumor segmentation (Pinto et al., 2016; Zhao and Jia, 2016) to name a few. In dementia research, several studies have investigated brains of patients with Alzheimer's disease (AD) using deep learning and shown impressive diagnostic abilities (Hosseini-Asl et al., 2016; Payan and Montana, 2015; Suk et al., 2016; Liu et al., 2018). A Recurrent Neural Network (RNN) is an artificial neural network that has an internal state (or “memory”) and is useful when processing sequential data, such as words in a sentence or frames in a video (Lecun et al., 2015; Donahue et al., 2015). RNN's have previously been combined with CNN's to segment MRI images, where the addition of an RNN module helped to leverage adjacent slice dependencies (Ypsilantis and Montana, 2016; Poudel et al., 2017).

In this study, we aimed to develop an automatic algorithm based on convolutional and recurrent neural networks that provides fast, reliable, and systematic predictions of established visual ratings scales of atrophy of brain regions often affected in dementia: the MTA, GCA-F and PA scales. The models are trained on a large set of MRI images that have been rated by an experienced neuroradiologist. This method is atlas-free and requires minimum amount of setup and third-party software. We plan to present the proposed algorithm as a freely available software targeted towards neuroimaging researchers.

## 2 Material and methods

### 2.1 MRI data and protocols

Two different dementia cohorts of MRI images were included in this project: Alzheimer's Disease Neuroimaging Initiative (ADNI) and a clinical cohort with images from the memory clinic at Karolinska University Hospital (referred to as *MemClin* from here on). Informed consent was obtained for all participants, or by an authorized representative of theirs.

Individuals in the MemClin cohort consisted of patients clinically diagnosed between 2003 and 2011 with AD according to the ICD-10 criteria, or frontotemporal dementia (FTD) using the diagnostic criteria by Neary et al. (1998). Brain images of these patients had been visually rated by neuroradiologist Lena Cavallin (L.C.) in previous studies by our group focused on AD and FTD, with the exclusion criteria of patients having history of traumatic brain injury (<1%) or insufficient quality of the MRI scan (<1%, not possible to visually rate) (Ferreira et al., 2018; Lindberg et al., 2009). An additional exclusion criterion in this study was failed automatic registration using FSL of images possible for a radiologist to visually rate (2.6%). All participants underwent a T_1_-weighted MRI scan at the Radiology Department of Karolinska University Hospital in Stockholm, Sweden.

Data used in the preparation of this article were obtained from the Alzheimer's Disease Neuroimaging Initiative (ADNI) database (adni.loni.usc.edu). The ADNI was launched in 2003 as a public-private partnership, led by Principal Investigator Michael W. Weiner, MD. The primary goal of ADNI has been to test whether serial magnetic resonance imaging (MRI), positron emission tomography (PET), other biological markers, and clinical and neuropsychological assessment can be combined to measure the progression of mild cognitive impairment (MCI) and early Alzheimer's disease (AD). For up-to-date information, see www.adni-info.org. A majority of the participants in the ADNI cohort were scanned multiple times within a few weeks—often in the same day. A subset of participants were scanned both in 1.5 T and 3 T machines.

All available images with an associated visual atrophy rating performed by a neuroradiologist were used in this study: a total of 5271 images. They were obtained from 117 different scanners (112 in ADNI and 5 in MemClin, see Supplementary Data for detailed description of scanner protocols), where all except 36 scans were acquired using a 3D MRI protocol. The MemClin image data was collected as part of the clinical routine with standard—but not strictly harmonized—MRI protocols, including updates and protocol changes over time. Thus, the MemClin data largely resembles the MRI variability present in clinics, whereas the ADNI data does not due to extensive efforts in harmonizing the MRI protocols across scanners.

We used theHiveDB database system (Muehlboeck et al., 2014) for data management during the development of the algorithm, which will become part of theHiveDB's automated activity system.

### 2.2 Human ratings

An experienced neuroradiologist (L.C.) visually rated 2350 T_1_-weighted MRI images over the course of 16 months with no prior knowledge of age, sex, or diagnosis. For ADNI subjects scanned more than once within a few weeks (i.e. within the same ADNI time-point), only one of these images was rated by the radiologist and the additional image(s) were labeled with the same rating. The distribution of L.C.'s MTA, PA and GCA-F ratings are shown in Table 2. Many of the ADNI ratings have been analyzed and reported in previous studies (Ferreira et al., 2018; Ferreira et al., 2015; Ferreira et al., 2016; Ferreira et al., 2017; Westman et al., 2019). All visual ratings of MTA, PA and GCA-F were based on T_1_-weighted MRI images, and illustrative examples of the ratings can be seen in Fig. 1. The images were aligned with AC-PC (the anterior and posterior commissures) by the radiologist if the protocol allowed for it (Cavallin et al., 2012a). The MTA ratings were made in a single coronal slice, just behind the amygdala and mammillary bodies. The GCA-F ratings were based on multiple axial slices, whereas the PA scores were based on slices in all three planes.

**Table 2:** The rating distribution of the images used in the study. The “Images” column refers to how many *unique* images that were rated by the radiologist at least once. Both the left and right MTA ratings are presented in the “MTA” column in the Table.

Cohort	Images	MTA					GCA-F				PA			
		0	1	2	3	4	0	1	2	3	0	1	2	3
ADNI	1966	425	1581	1147	555	224	1449	468	49	0	1188	611	157	10
MemClin	384	23	265	296	139	45	279	89	14	2	210	127	43	4
Total	2350	448	1846	1443	694	269	1728	557	63	2	1398	738	200	14

To get an idea of the variability in the human ratings used for training AVRA, we studied the intra-rater agreement in a subset of 244 images that had been rated 2–4 times with at most 16 months from the first to the last rating session by L.C. To be consistent with the computer training and evaluation procedure, we compared the latest rating to a previous one. If there were more than two ratings, the previous rating was chosen randomly.

### 2.3 Computer ratings

The motivation behind the proposed model architecture was to mimic how a neuroradiologist would process an MRI image: to scroll through the brain volume slice-by-slice looking for the “correct” slice(s) to base the rating on. A human rater assesses images acquired using different scanners, vendors and protocols without any need for substantial preprocessing such as segmentation, intensity normalization, non-linear registrations or skull-stripping. To better mimic the clinical situation (and to keep the number of time consuming preprocessing steps that can potentially fail to a minimum) we trained AVRA to rate images with as little preprocessing as possible. The main difference between AVRA's and a human rater is that AVRA's ratings are continuous instead of discrete.

All code in this project was developed in Python 3.4.3 using the deep learning framework PyTorch 1.0 (Paszke et al., 2017). The training of AVRA was done on GPU's for computational efficiency. To obtain an estimate of the time to run a single case when run on a “regular” computer, we processed and timed five individual cases using CPU only (Intel®Core ™i7-8700k, 32GB DDR-4) without GPU support.

#### 2.3.1 Preprocessing

**Fig. 2:**
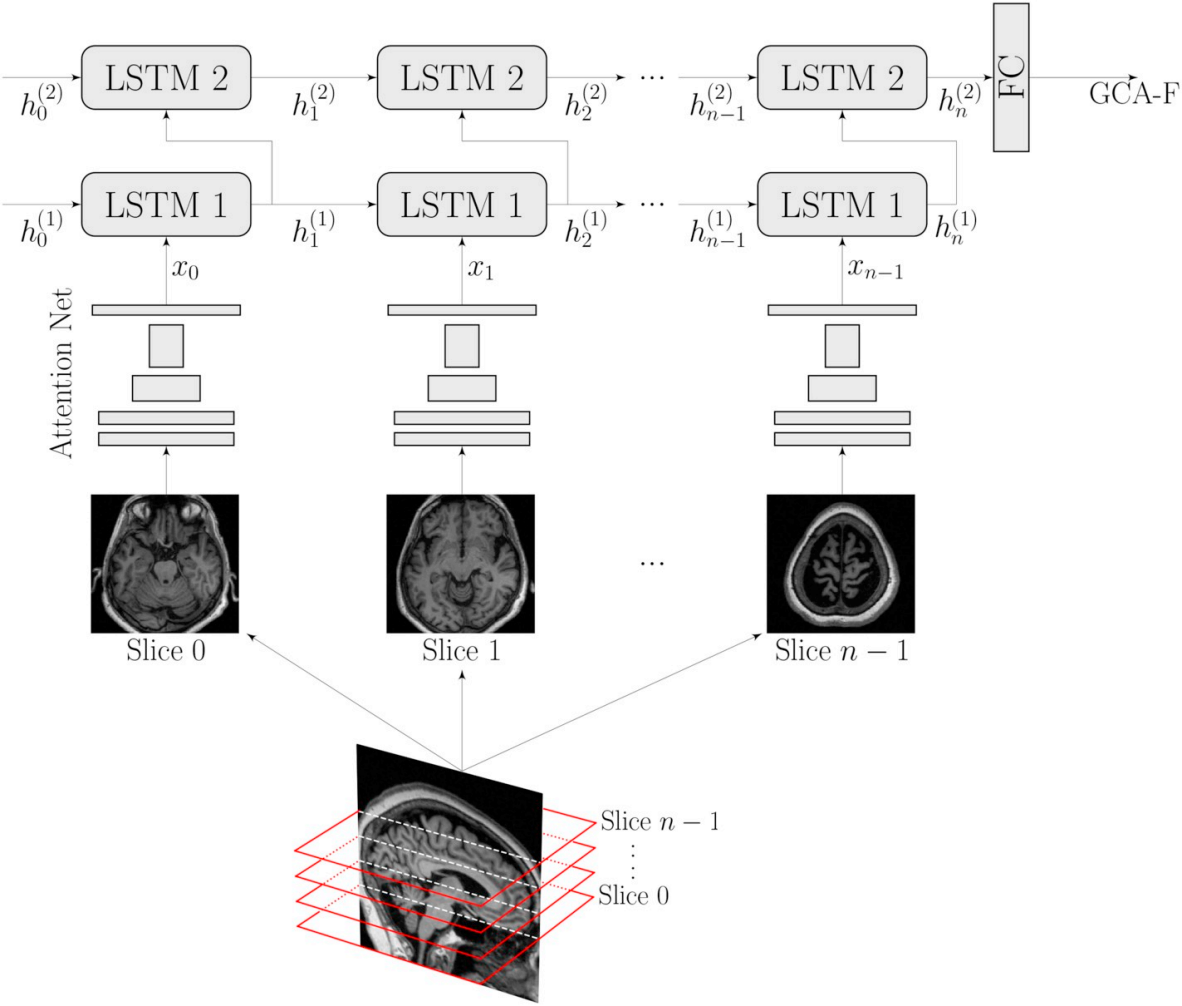
A sketch of the architecture of AVRA, with the example of a GCA-F prediction. Briefly, each slice is processed through the residual attention network and the extracted features are passed to a 2-layer Long-Short Term Memory (LSTM) network. Once all MRI slices have propagated through these two stages, a fully connected network (FC) makes a prediction of the visual atrophy rating. The MTA and PA models followed the same structure.

The only preprocessing included in our method is the registration of all brains to the MNI standard brain using FSL FLIRT 6.0 (FMRIB's Linear Image Registration Tool) (Jenkinson et al., 2002; Jenkinson and Smith, 2001; Greve and Fischl, 2009). This rigid transform is computed with 6 degrees of freedom (i.e. rotation and translation only) and is used to automatically AC-PC align each brain and conform all images to the same voxel size (1x1x1mm^3^) and input dimension (182x218x182). The AC-PC aligned images are cropped to remove excess space outside the brain and redundant slices not part of the ratings scale (as indicated in Fig. 1, Fig. 2). The center-voxel of the cropped images depended on the rating scale. For the MTA ratings, 22 coronal slices of the dimension 128 mm × 128 mm are input to the model—enough to ensure that the “correct” rating slice is included. The GCA-F ratings are done on multiple axial slices so each volume is cropped to 160 mm × 192 mm × 40 slices, with 2 mm slice thickness. The PA model requires slices from all three anatomical planes. From each MRI image a smaller volume of 128 mm × 128 mm × 128 mm was extracted from the parietal lobe, sufficiently large to include all relevant structures in the parietal cortex. From this cropped volume 37 axial, 28 coronal and 34 sagittal slices with 2 mm slice thickness (i.e. 99 slices in total) were used as input to the model. Since the distribution of raw voxel values was very different—particularly between 1.5 T and 3 T images—all cropped volumetric images were normalized to have a zero variance and mean.

#### 2.3.2 Model architectures

**Fig. 3:**
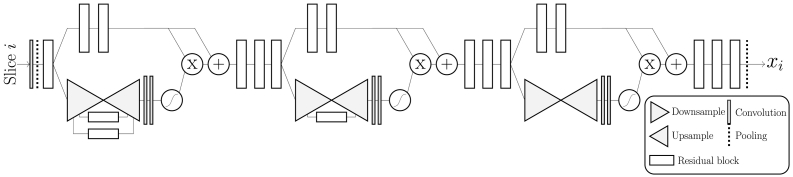
A sketch of the residual attention net used to extract features from individual MRI slices, where the flattened output is fed to the RNN. The downsampling block consists of stacking maxpooling operations followed by a residual block. The upsamling is performed with bilinear interpolations of the output of a residual block. The “+”, “x”, and “S-shaped” symbols denote element-wise summation, multiplication and the sigmoid function, respectively. The flow chart is adapted from (Wang et al., 2019).

The overall structure of the models is shown in Fig. 2 and can be split into three parts. First, relevant features from a single slice are extracted using a Residual Attention Network (Wang et al., 2019), detailed in Fig. 3. It combines the abilities from residual learning (He et al., 2016), which can allow for even deeper models, and attention models that can “focus” spatially on images—particularly useful for visual ratings since they are based on regional atrophy (Xu et al., 2015; Ba et al., 2015). Our implementation is a slimmed version of the original, with the same depth but a smaller number of filters in each layer to reduce memory usage and computation time. Initial experiments showed no noticeable performance reduction on the validation set compared to using a larger network. Second, the features are reshaped to a 1D vector and fed to an RNN, which consists of a two-layer Long-Short Term Memory (LSTM) network with 256 hidden nodes (Hochreiter and Schmidhuber, 1997; Gers and Cummins, 2019). The LSTM modules are expected to “remember” relevant features seen in previous slices and update its state (“memory”) when it is exposed to a slice containing useful information for the rating. Finally, when slice 0, 1, …, (*n* − 1) have been propagated through the network, the final output from the second LSTM module *h*_*n*_^(2)^ is used to make a linear prediction of the visual rating. All three models share the same network architecture except for the size of the input vector fed to the LSTM network, as that is dependent on the input size of the MRI slices.

For comparison, we trained a VGG16 network (Simonyan and Zisserman, 2019) without the RNN part, where the 3D volumes are treated as multi-channel 2D images. That is, for the MTA model we input one “22-channel” image to the CNN once instead of 22 single-slice images.

#### 2.3.3 Training

For training and evaluation, the dataset was randomly split into a training and a hold-out test set, where 20% of all subjects were assigned to the test set. On the remaining images in the training set we applied 5-fold cross validation for hyper-parameter tuning for each rating scale. The five trained models were used together as an ensemble classifier evaluated on the test set, where the average prediction was considered the final rating.

The models were trained for 200 epochs using backpropagation and optimized through stochastic gradient descent (SGD) with cyclic learning rate to maximize the probability of predicting the radiologist's rating (Loshchilov and Hutter, 2016; Huang et al., 2017). The training set was randomly split into minibatches, each containing 20 MRI images, and the weights were updated to minimize the mean-squared error between the automatic and the integer ratings by L.C. We employed data augmentation in the training process of the network to reduce the risk of overfitting to the training set. This included random cropping (within ±10mm off the center voxel), scaling, left/right mirroring, and randomly selecting N4ITK inhomogeniety corrected images instead of the original file (Tustison et al., 2010). Due to the imbalance of ratings in the dataset we employed random oversampling of images with less frequent ratings, which has been shown to improve the prediction performance of CNN's (Buda et al., 2017). For ADNI subjects that had multiple scans for a single timepoint, a scan was selected randomly for each minibatch.

### 2.4 Analyses metrics

The visual rating scales are subjective measures by definition. Consequently, there are no objective ground truth ratings available. In most studies, the performance of a rater is reported in kappa statistics—a group of measures that can quantify the level of agreement between two sets of discrete ratings—but there is no single metric always reported. To make our results comparable to previous findings, we present our results with Cohen's weighted kappa (*κ*_*w*_), which has been used in several previous rating studies (Koedam et al., 2011; Westman et al., 2019; Cavallin et al., 2012a; Cavallin et al., 2012b; Ferreira et al., 2016; Ferreira et al., 2017; Velickaite et al., 2017), as well as accuracy and the Pearson correlation coefficient (*ρ*). The agreement between two sets of ratings is referred to as *inter*-rater agreement if the sets were assessed by different raters, and *intra*-rater agreement if a single radiologist rated the set twice.

## 3 Results

For the 244 images rated more than once by the radiologist the intra-rater agreement *κ*_*w*_ and accuracy for MTA (left) were: *κ*_*w*_ = 0.83, acc = 76%; MTA (right): *κ*_*w*_ = 0.79, acc = 70%; GCA-F: *κ*_*w*_ = 0.46, acc = 71%; PA *κ*_*w*_ = 0.65, acc = 72%. Ratings made only 1 week apart showed substantially better intra-rater agreement (see *Ferreira* et al. *(2017)* entry in Table 3). These results provide an estimate of the “human-level agreement”—i.e. approximate levels of agreement our models should be able to achieve by training on the available cohort due to rating inconsistencies over 16 months. Since there are no random elements in the evaluation process of a brain image, the “intra-rater” agreement of AVRA is inherently *κ*_*w*_ = 1.

**Table 3:** Previous studies reporting weighted kappa (*κ*_*w*_) values for intra- and inter-rater agreements together with the test set agreement between L.C. and AVRA (in bold text), and L.C and VGG16 as a reference. The interval given refers to the minimum and maximum *κ*_*w*_ value reported in the referenced study. The *N* column refers to the number of images used for the intra- and inter-rater assessment (if two values are given the number of images rated were different for the intra- and the inter-rater analysis). ^∗^ denotes if L.C. (whose ratings was used for training in this study) was one of the raters in the reported agreements.

Study	Scale	*N*	Intra-rater agreement (*κ*_*w*_)	Inter-rater agreement (*κ*_*w*_)
Cavallin et al. (2012)	MTA	100	0.83–0.94^∗^	0.72–0.84^∗^
Cavallin et al. (2012b)	MTA	100	0.84–0.85^∗^	–
Westman et al. (2011)	MTA	100	0.93^∗^	–
Velickaite et al. (2017)	MTA	20/50	0.79–0.84	0.6–0.65^∗^
Ferreira et al. (2017)	MTA	120	0.89–0.94^∗^	0.70–0.71^∗^
Koedam et al. (2011)	MTA	29/118	0.91–0.95	0.82–0.90
VGG16	MTA	464	1	0.58–0.59^∗^
**AVRA**	**MTA**	**464**	**1**	**0.72–0.74**^∗^
Koedam et al. (2011)	PA	29/118	0.93–0.95	0.65–0.84
Ferreira et al. (2017)	PA	120	0.88^∗^	0.88^∗^
VGG16	PA	464	1	0.63^∗^
**AVRA**	**PA**	**464**	**1**	**0.74**^∗^
Ferreira et al. (2016)	GCA-F	100	0.70^∗^	0.59^∗^
Ferreira et al. (2017)	GCA-F	120	0.83^∗^	0.79^∗^
VGG16	GCA-F	464	1	0.56^∗^
**AVRA**	**GCA-F**	**464**	**1**	**0.62**^∗^

Our models predicted continuous rating scores of an image, based on training from discrete ratings by L.C. We rounded AVRA's ratings to the nearest integer to be able to compare the rating consensus in terms of accuracy and kappa statistics. The agreements between the radiologist's and AVRA's (as well as the VGG networks') ratings on the hold-out test set are summarized in Table 3 together with previously reported *κ*_*w*_ values of inter- and intra-rater agreements. The inter-rater agreement *k*_*w*_, Pearson correlation *ρ*, and accuracy on the test set for MTA (left): *κ*_*w*_ = 0.74, *ρ* = 0.88, acc = 70%; MTA (right): *κ*_*w*_ = 0.72, *ρ* = 0.88, acc = 70%; GCA-F: *κ*_*w*_ = 0.62, *ρ* = 0.71, acc = 84%; PA: *κ*_*w*_ = 0.74, *ρ* = 0.85, acc = 83%. These agreement levels were similar to previously reported in studies, see Table 3. The naive VGG16 implementations showed lower inter-rater agreements with the radiologist compared to AVRA.

**Fig. 4:**
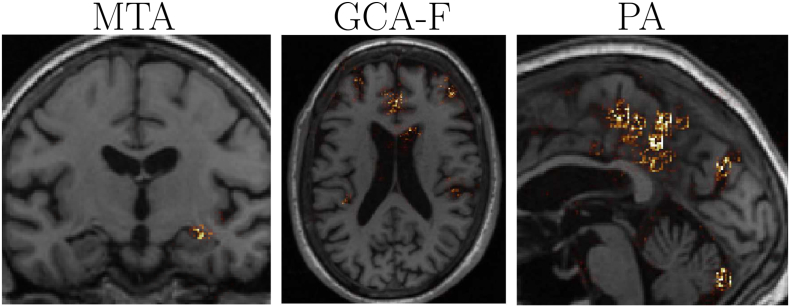
Examples of sensitivity maps for the MTA, GCA-F and PA scale, respectively. These maps indicate the influence each voxel had in AVRA's rating. The particular slices displayed were chosen manually as representative images for each rating scale.

To increase interpretability and understanding of the models, we computed gradient-based sensitivity maps of images in the test set based on the SmoothGrad method (Smilkov et al., 2019). These indicated how influential individual voxels were in the rating prediction, which we can apply to verify that the network identified the correct features. Examples of AVRA's rating predictions for each scale are shown in Fig. 4. As can be observed, the MTA sensitivity maps were generally focused only around the area of the hippocampus and the inferior lateral ventricle in ∼± 3 slices from the “correct” rating slice. The sensitivity maps in other more posterior and anterior slices were close to zero. The GCA-F maps were more diffused, but the greatest magnitudes were primarily seen in the sulci of the frontal lobe. The PA maps were mainly visible in the parietal lobe and in the sagittal plane, with the greatest magnitudes appearing in parieto-occiptal sulcus and precuneus.

The average time to process a single image using AVRA without GPU support was 48 s, where the majority of the processing time was spent on the AC-PC alignment using FSL FLIRT.

## 4 Discussion

We have developed a tool for automatic visual ratings of atrophy (AVRA) that is fast, systematic and robust. AVRA is trained on a large set of images rated by an expert neuroradiologist using the established clinical assessment measures of Scheltens' MTA scale, Pasquier's GCA-F scale and Koedam's PA scale with agreement levels similar to that between two experienced radiologists. This tool runs in under 1 min on a regular computer, which enables automatically rating thousands of images in a couple of hours. The main advantage of an automatic model is the absence of randomness, which can improve rating consistency between different clinics, research groups and cohorts. Thus, AVRA can increase the use of visual ratings in research, and has—after extensive validation—the potential to function as a clinical aid in the future.

The rating agreements between AVRA's and the radiologist's ratings were considered *substantial* (i.e. between 0.6 and 0.8) according to the often cited paper by Landis and Koch (1977) (Landis and Koch, 1977). The agreements were close to the “human-level agreements” in this study (i.e. the agreement between the multiple L.C. ratings of the same image). This was reasonable since a model trained on imperfect labels due to rating inconsistency can never achieve perfect agreement. AVRA's ratings agreed more with the radiologist ratings than the VGG16 models' did. A recurrent CNN architecture might thus be particularly suitable for visual rating predictions, but we can not say from these results if it was the residual modules, the attention components, or the LSTM cells—all used in AVRA but not in the VGG16 models—that had the greatest positive impact on the performance. Another contributing factor may be the wide difference in the number of trainable parameters between AVRA (1.5 M) and VGG16 (65 M) that makes AVRA less prone to overfit on the training data.

The automatic model presented by Lötjönen and colleagues (2017) is, to our knowledge, the only software that also attempts to predict scores based on clinical visual rating scales (Lotjonen et al., 2017). It is based on volume measures of hippocampus and surrounding structures, whereas AVRA predicts the ratings directly from the voxel intensity values. This makes our proposed method promising to also work on MRI images with large slice thickness and CT images, from which volumes generally cannot be computed. The fact that CT is a cheaper and more commonly used imaging modality than MRI in the clinics speaks in favor of using convolutional neural networks over volumetrics for automatic predictions of visual ratings (Falahati et al., 2015). No *κ*_*w*_ values are reported in (Lotjonen et al., 2017), but they provided correlation coefficients between radiologist and computer ratings for the MTA scale as 0.86 (left) and 0.85 (right). AVRA showed a similar magnitude of correlation for the MTA scale on the hold-out test set: *ρ* = 0.88.

Frequently, it is difficult for a radiologist to decide between two scores, and in a clinical situation the level of atrophy is often described as “the left MTA is between 2 and 3” for instance. This nuance might be important information for the physician diagnosing dementia, but in research single integer scores have typically been used following the original definitions of the rating scales. Previous attempts of (semi-)automatic atrophy measures have output a continuous measure (Zimny et al., 2013; Menéndez-González et al., 2014; González et al., 2019; Lotjonen et al., 2017). The main advantages of using a continuous measure of atrophy are 1) atrophy evolves continuously and thus it is reasonable to describe its degree through a continuous measure, and 2) it provides more detailed information about the severity of the atrophy. The latter point is for instance particularly useful to track disease progression and could allow us to establish more sensitive cut-off values for different diagnoses.

**Fig. 5:**
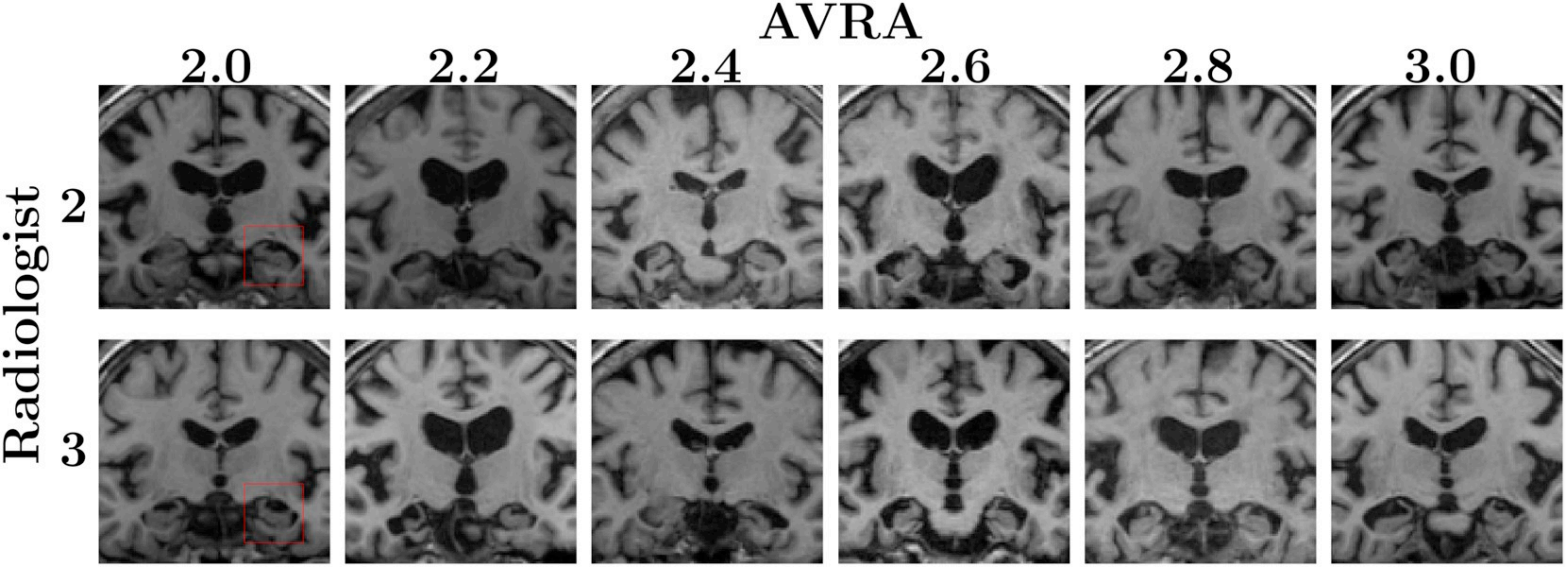
Comparison between AVRA's continuous ratings and the neuroradiologist's discrete ratings of the same images. *Rows*: MRI slices with MTA on the right side of the image (side indicated by the red squares) rating of 2 (top) and 3 (bottom) given by neuroradiologist. *Columns*: corresponding continuous AVRA ratings. E.g., the second image from the left in the bottom row was given assessed to have a left MTA score of 3 by the neuroradiologist and 2.2 by AVRA. When the radiologist re-examined these cases the same ratings were given for all images, except for the three images on the right in the top row (Radiologist: 2, AVRA: 2.6, 2.8 and 3.0), which were instead given MTA scores of 3. The image rated 2 by L.C. and 2.4 by AVRA was described as a subject between 2 and 3.

In Fig. 5 we show some examples between AVRA's continuous and the radiologist discrete ratings in the important diagnostic interval between MTA = 2 and MTA = 3. When studying these images again post AVRA's ratings, the radiologist only assessed that the images originally rated MTA = 2 with associated AVRA scores of 2.6–3.0 to be wrongly rated. They would be re-rated as MTA = 3, i.e. closer to AVRA's score. The image scored MTA = 2 (radiologist) and MTA = 2.4 (AVRA) was described as a case between 2 and 3, which may illustrate the usefulness of continuous ratings. However, we noticed that in two of the most disagreeing ratings (L.C.: MTA = 3, Avra: MTA = {2.0, 2.2}) the individuals had an adhesion between the hippocampus and the cerebral white matter. These cases are not frequent, and the rating disagreements in Fig. 5 indicate that AVRA did not learn to correctly adjust the score for the presence of adhesions.

One of the main motivations of having a computer rate brain atrophy instead of humans is its inherent perfect intra-rater agreement—the same image will be rated exactly the same regardless of when (and where) it is rated. A relevant question to ask is: why not let a computer segment and calculate e.g. hippocampal volumes instead of an MTA rating? We see two main motivations for this: First, CT, and some MRI protocols, have too large slice thickness that do not allow for extracting reliable volumetric information from the images. While not explicitly investigated in this project, the RNN component of AVRA (allowing us to extract information from the MRI slice-by-slice) makes it possible to process images with large slice thickness. Second, segmentation methods will—just as AVRA—fail in processing some cases, and for clinician to manually intervene and delineate structures would neither be feasible nor practical. If an automatic visual rating would fail the radiologist would be able to quickly perform their own visual rating, as is done today.

The sensitivity maps shown in Fig. 4 suggested that the models were able to correctly identify relevant structures to base their ratings on. Particularly the sensitivity maps of the MTA model were typically not visible ±3mm from the “correct” rating slice, indicating that the employed recurrent CNN architecture used was able to correctly identify relevant slices and disregard redundant ones. The diffused sensitivity maps seen for the GCA-F scale was also observed in the quantitative validation study using surface-based analysis by Ferreira et al. (2015), showing that frontal atrophy is also associated with temporal and posterior atrophy—at least in the ADNI cohort (Ferreira et al., 2016). Möller et al. (2014) found, using VBM analysis, significant differences between PA ratings not only in the parietal lobe, but also in parts of the cerebellum, temporal lobe and the occipital lobe (Möller et al., 2014). Their study was also performed on a cohort with individuals with probable AD and subjective memory complaints, concluding that atrophy solely in the posterior cortex is an exception. The sensitivity maps from our PA model indicate that AVRA based the PA ratings on mainly the same regions. AVRA learns to how to predict a GCA-F or a PA score from an MRI image *only* based on previous human ratings. Thus, if e.g. frontal atrophy is strongly associated with atrophy in the temporal lobe, the model is likely to find it difficult to learn to only assess the frontal lobe in the GCA-F scale.

**Fig. 6:**
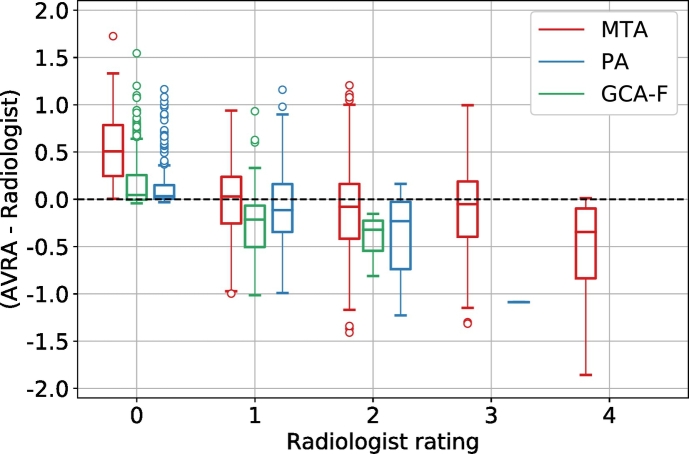
Box plots of the difference between AVRA's continuous and the radiologist's discrete ratings of the same image (stratified by radiologist score) for the MTA (red), GCA-F (green), and the PA scale (blue). There were no images assigned a rating of GCA-F = 3 by the radiologist and only 1 image with PA = 3 in the test set, which explains the absence of boxes for these ratings.

There are some limitations of the proposed algorithm. First, the models are solely based on the ratings by a single radiologist and thus assume that the ratings we trained the model on are “ground truth” labels. A model trained on these labels can therefore never be “better” than the rater. If the ratings have systematic errors the model will incorporate these. For instance, a rater might systematically look at the left medial temporal lobe when rating the MTA of the right hemisphere, which could influence (bias) the right hemisphere MTA score. If we train a model on these ratings, this bias would be learned by the model as well. Another approach would be to have multiple expert radiologists rate a set of images together or separately and use these labels as ground truth. However, it is not feasible to have multiple radiologist visually assess the large number of images necessary for training a deep neural network. If future studies want to use a neural network based on their own set of ratings, it should be possible to start from the pre-trained networks of AVRA and fine-tune the final classification layer(s) on the new ratings. This would require substantially fewer ratings, since the convolutional part would already have learned to extract relevant features from the images. The second limitation of the study are the small numbers of the highest GCA-F and PA ratings, which may increase the risk of “true” 3 score to be misclassified. Based on the results in Fig. 6 this seems to be the case. As the diagnostic cut-off values for these ratings scales in AD diagnosis have been suggested as PA ≥ 1 and GCA-F ≥ 1 (Ferreira et al., 2015), the clinical implications of this may be minor even in the cases where the atrophy is rated as a 2 instead of a 3. These severe ratings are rare also in previous studies on dementia cohorts (Ferreira et al., 2015; Rhodius-Meester et al., 2017), so this will likely be an issue for any computerized method trained on radiologist ratings.

The performance of AVRA was validated in a test set that was randomly sampled from the same cohorts as the training data set. This is a simpler test set than if the test set was from a different cohort with images acquired using other scanning parameters, which would better reflect the clinical setting. Further, since AVRA is evaluated on scores by the same radiologist that rated the training set, the agreement levels are expected to be lower if compared to an external rater. We are currently in the process of validating how the models would handle data acquired with different MRI protocols and the effect it would have on rating agreement.

## 5 Conclusion

In this study, we have proposed an automatic method (AVRA) to provide visual ratings of atrophy according to Scheltens' MTA scale, Koedam's PA scale, and Pasquier's frontal GCA scale. AVRA mimics the neuroradiologist's rating procedure and achieves similar levels of agreement to that between two experienced neuroradiologists—without any prior preprocessing of the MRI images. We plan to make AVRA freely available as a user-friendly software aimed towards neuroscientists.

## Appendix A Supplementary data

Supplementary material Image 1
