# Supplementary material for: AVRA: Automatic visual ratings of atrophy from MRI images using recurrent convolutional neural networks
Source: Neuroimage Clin. 2019 May 25;23:101872. doi: 10.1016/j.nicl.2019.101872 (PMC6545397; doi:10.1016/j.nicl.2019.101872)
Supplement: Supplementary file 1 — Supplementary material [file mmc1.pdf]

Supplementary Data - AVRA: Automatic Visual Ratings of Atrophy from MRI images using Recurrent Convolutional Neural Networks.

Gustav Mårtensson[a,*], Daniel Ferreira[a], Lena Cavallin[b,c], J-Sebastian Muehlboeck[a], Lars-Olof Wahlund[a], Chunliang Wang[d], Eric Westman[a,e], for the Alzheimer's Disease Neuroimaging Initiative [☆]

[a] *Division of Clinical Geriatrics, Department of Neurobiology, Care Sciences and Society, Karolinska Institutet, Stockholm, Sweden.*
[b] *Department of Clinical Neuroscience, Karolinska Institutet, Stockholm, Sweden.*
[c] *Department of Radiology, Karolinska University Hospital, Stockholm, Sweden.*
[d] *School of Technology and Health, KTH Royal Institute of Technology, Stockholm, Sweden.*
[e] *Department of Neuroimaging, Centre for Neuroimaging Sciences, Institute of Psychiatry, Psychology and Neuroscience, King's College London, London, UK.*

1. MRI scanning protocols

Table 1 provides a summary of the MRI protocols used for the images in this project. Table 2 and 3 shows a a detailed table of each individual scanner and the scanning parameters used to acquire the images for the MemClin and ADNI cohort, respectively.

Table 1: Summary statistics of the two cohorts used in the project, stratified by field strength. The entries shows the minimum and maximum values in each column as "min - max". Abbreviations: Field strength (FS); Echo time (TE); Repetition time (TR); Inversion recovery time (IT); Slice thickness (ST).

**Cohort: FS**	**N**	**TE** (ms)	**TR** (ms)	**IT** (ms)	**ST** (mm)
ADNI: 1.5T	3996	2.88 - 4.96	8.51 - 3000	1000	1.2
MemClin: 1.5T	327	2.56 - 4.4	11.4 - 2400	300 - 1100.0	1.2 - 2.5
ADNI: 3.0T	837	2.84 - 3.25	6.39 - 2300.0	0.0 - 1000.0	1.2
MemClin: 3.0T	57	2.57 - 3.42	1780.0 - 2300.0	900.0	1.0 - 1.4

Table 2: Detailed MRI protocols from MemClin cohort, where each row represent an individual scanner. Abbreviations: Echo time (TE); Repetition time (TR); Inversion recovery time (IT); Slice thickness (ST); Resolution in x-y-plane (x-y, equal lengths).

**Scanner**	**N**	**FS** (T)	**TE** (ms)	**TR** (ms)	**IT** (ms)	**x-y** (mm)	**ST** (mm)
Siemens Aera	2	1.5	3.18	2300	904	1.25	1.20
Siemens Avanto	204	1.5	2.56 - 4.19	1160 - 2400	600 - 1100	0.45 - 1.30	1.20 - 2.50
Siemens Magnetom vision	36	1.5	4.40	11.40	300	0.90	2.50 - 2.50
Siemens Symphony	85	1.5	3.93	1960	790 - 875	0.49	1.41 - 1.51
Siemens Triotim	57	3.0	2.57 - 3.42	1780 - 2300	900	0.85 - 1	1 - 1.40

[☆] Data used in preparation of this article were obtained from the Alzheimer's Disease Neuroimaging Initiative (ADNI) database (adni.loni.usc.edu). As such, the investigators within the ADNI contributed to the design and implementation of ADNI and/or provided data but did not participate in analysis or writing of this report. A complete listing of ADNI investigators can be found at: http://adni.loni.usc.edu/wp-content/uploads/how_to_apply/ADNI_Acknowledgement_List.pdf.

[*] Corresponding author
Email address: gustav.martensson@ki.se (Gustav Mårtensson)

Table 3: Detailed MRI protocols from ADNI cohort, where each row represent an individual scanner. Abbreviations: Echo time (TE); Repetition time (TR); Inversion recovery time (IT); Slice thickness (ST); Resolution in x-y-plane (x-y).

**Scanner**	**N**	**FS** (T)	**TE** (ms)	**TR** (ms)	**IT** (ms)	**x-y** (mm)	**ST** (mm)
GE Genesis signa	114	1.5	4.10	10.20	1000	0.94	1.20
GE Genesis signa	16	1.5	4.08 - 4.10	10.20	1000	0.94 - 1.02	1.20
GE Genesis signa	18	1.5	4.09	10.40	1000	0.94	1.20
GE Genesis signa	39	3	3.05	7.50	900	1.02	1.20
GE Genesis signa	4	1.5	4.09	10.20	1000	0.94	1.20
GE Genesis signa	48	1.5	4.08 - 4.10	10.20	1000	0.94 - 1.09	1.20
GE Genesis signa	89	1.5	4.09	10.40	1000	0.94	1.20
GE Signa excite	10	3.0	2.84 - 2.85	6.61 - 6.63	900	1.02	1.20
GE Signa excite	107	1.5	3.92 - 4.05	8.90 - 9.20	1000	0.94 - 1.02	1.20
GE Signa excite	14	1.5	3.80 - 3.90	8.59 - 8.81	1000	0.94	1.20
GE Signa excite	155	1.5	3.80 - 3.92	8.59 - 8.99	1000	0.94 - 0.98	1.20
GE Signa excite	158	1.5	3.92 - 4.05	8.92 - 9.20	1000	0.94	1.20
GE Signa excite	17	1.5	3.96	9.12	1000	0.94	1.20
GE Signa excite	2	1.5	3.96	9.12	1000	0.94	1.20
GE Signa excite	21	1.5	3.80	8.59	1000	0.94	1.20
GE Signa excite	24	3.0	2.84 - 2.85	6.62 - 6.63	900	1.02	1.20
GE Signa excite	30	1.5	3.92 - 4.06	8.92 - 9.22	1000	0.94	1.20
GE Signa excite	34	3.0	2.84 - 2.99	6.61 - 7.04	900	1.02	1.20
GE Signa excite	39	1.5	3.80 - 3.91	8.60 - 8.84	1000	0.94	1.20
GE Signa excite	45	1.5	3.96	9.12	1000	0.94	1.20
GE Signa excite	46	1.5	3.80 - 3.90	8.59 - 8.81	1000	0.94	1.20
GE Signa excite	51	1.5	3.92 - 4.05	8.92 - 9.20	1000	0.94	1.20
GE Signa excite	52	1.5	3.92 - 3.94	8.92 - 8.94	1000	0.94	1.20
GE Signa excite	56	1.5	3.78 - 3.98	8.56 - 9.12	1000	0.94 - 1.02	1.20
GE Signa excite	60	1.5	3.79 - 3.91	8.58 - 8.84	1000	0.94 - 1.02	1.20
GE Signa excite	8	3.0	2.86 - 3.01	6.64 - 6.96	900	1.02	1.20
GE Signa excite	84	1.5	3.92 - 3.96	8.92 - 9.12	1000	0.94	1.20
GE Signa excite	88	1.5	3.80 - 3.90	8.59 - 8.81	1000	0.94	1.20
GE Signa excite	9	3.0	2.84 - 2.99	6.62 - 6.91	900	1.02	1.20
GE Signa excite	93	1.5	3.96	9.12	1000	0.94	1.20
GE Signa excite	95	1.5	3.78 - 3.91	8.57 - 8.84	1000	0.94	1.20
GE Signa hdx	10	3.0	2.84 - 2.85	6.61 - 6.63	900	1.02	1.20
GE Signa hdx	106	1.5	3.80	8.59	1000	0.94	1.20
GE Signa hdx	149	1.5	3.79 - 4.96	8.58 - 11.05	1000 - 1044	0.94 - 1.09	1.20
GE Signa hdx	16	1.5	3.80 - 3.90	8.59 - 8.81	1000	0.94	1.20
GE Signa hdx	20	1.5	3.80 - 3.90	8.59 - 8.81	1000	0.94	1.20
GE Signa hdx	24	1.5	3.92	8.92	1000	0.94	1.20
GE Signa hdx	25	1.5	4.04	9.18	1000	0.94	1.20
GE Signa hdx	50	1.5	3.80	8.59	1000	0.94	1.20
GE Signa hdx	6	1.5	3.80	8.59	1000	0.94	1.20
GE Signa hdx	92	1.5	3.80 - 3.92	8.59 - 8.99	1000	0.94	1.20
GE Signa hdx	92	1.5	3.97 - 4.05	9 - 9.20	1000	0.94	1.20
GE Signa hdxt	2	1.5	3.80	8.60	1000	0.94	1.20
GE Signa hdxt	6	3.0	2.84 - 2.85	6.61 - 6.63	900	1.02	1.20
Philips Achieva	12	3.0	3.05 - 3.17	6.39 - 6.81	1000	1.00	1.20
Philips Achieva	24	3.0	3.25	6.85	1000	1.00	1.20
Philips Achieva	35	3.0	3.13 - 3.25	6.78 - 6.85	1000	1.00	1.20
Philips Achieva	65	1.5	4 - 4.01	8.62	1000	0.90 - 0.98	1.20
Philips Intera	10	3.0	3.16	6.80	0 - 1000	1.00	1.20
Philips Intera	17	3.0	3.16 - 3.18	6.80 - 6.92	0 - 1000	1.00	1.20
Philips Intera	2	3.0	3.16	6.80	0	1.00	1.20
Philips Intera	26	3.0	3.13 - 3.25	6.76 - 6.84	1000	1.00	1.20
Philips Intera	34	1.5	3.99 - 4.01	8.58 - 8.62	0 - 1000	0.94 - 0.98	1.20
Philips Intera	38	1.5	4	8.62	0 - 1000	0.94	1.20
Philips Intera	4	1.5	4	8.62	1000	0.94	1.20
Philips Intera	4	3.0	3.16	6.80 - 6.81	1000	1.00	1.20

Philips Intera	40	1.5	3.98 - 4.01	8.51 - 8.62	1000	0.94	1.20
Philips Intera	48	3.0	3.16	6.80 - 6.81	1000	1.00	1.20
Philips Intera	54	1.5	3.99 - 4.01	8.51 - 8.63	1000	0.94 - 0.98	1.20
Philips Intera	6	1.5	4 - 4.01	8.61	1000	0.94	1.20
Philips Intera	6	3.0	3.13 - 3.25	6.76 - 6.84	1000	1.00	1.20
Philips Intera	61	1.5	4	8.61 - 8.62	1000	0.94	1.20
Philips Intera	66	1.5	4	8.59 - 8.61	1000	0.94	1.20
Philips Intera achieva	4	1.5	3.98	8.55	1000	0.94	1.20
Siemens Allegra	12	2.9	2.91	2300	900	1.00	1.20
Siemens Allegra	54	2.9	2.91	2300	900	1.00	1.20
Siemens Allegra	72	2.9	2.91	2300	900	1.00	1.20
Siemens Avanto	109	1.5	3.50	2400	1000	1.25	1.20
Siemens Avanto	30	1.5	3.50	2400	1000	1.25	1.20
Siemens Avanto	30	1.5	3.52 - 3.54	2400	1000	1.25 - 1.30	1.20
Siemens Avanto	4	1.5	3.50	2400	1000	1.25	1.20
Siemens Avanto	44	1.5	3.50	2400	1000	1.25	1.20
Siemens Avanto	52	1.5	3.52 - 3.54	2400	1000	1.25 - 1.30	1.20
Siemens Avanto	63	1.5	3.50	2400	1000	1.25	1.20
Siemens Espree	8	1.5	3.59	2400	1000	1.25	1.20
Siemens Sonata	116	1.5	3.54	2400	1000	1.25	1.20
Siemens Sonata	117	1.5	3.54	2400	1000	1.25	1.20
Siemens Sonata	63	1.5	3.54	3000	1000	1.25	1.20
Siemens Sonata	72	1.5	3.54	3000	1000	1.25	1.20
Siemens Sonata	77	1.5	3.54 - 3.55	3000	1000	1.25	1.20
Siemens Sonatavision	2	1.5	3.57	3000	1000	1.25	1.20
Siemens Sonatavision	30	1.5	3.54	2400	1000	1.25	1.20
Siemens Symphony	100	1.5	3.61	3000	1000	1.25	1.20
Siemens Symphony	104	1.5	3.61 - 3.67	3000	1000	1.25	1.20
Siemens Symphony	2	1.5	3.61	3000	1000	1.25	1.20
Siemens Symphony	20	1.5	3.61	3000	1000	1.25	1.20
Siemens Symphony	29	1.5	3.61	3000	1000	1.25	1.20
Siemens Symphony	30	1.5	3.67 - 3.71	3000	1000	1.25 - 1.35	1.20
Siemens Symphony	58	1.5	3.61 - 3.79	3000	1000	1.25 - 1.35	1.20
Siemens Symphony	81	1.5	2.88 - 3.65	3000	1000	1.25	1.20
Siemens Symphony	92	1.5	3.87	3000	1000	1.25	1.20
Siemens Symphony	97	1.5	3.59 - 3.63	3000	1000	1.25 - 1.30	1.20
Siemens Symphonytim	110	1.5	3.64	3000	1000	1.25	1.20
Siemens Trio	140	3.0	2.94	2300	900	1.00	1.20
Siemens Trio	4	2.9	2.94	2300	900	1.00	1.20
Siemens Trio	44	2.9	2.94	2300	900	1.00	1.20
Siemens Trio	5	3.0	2.94	2300	900	1.00	1.20
Siemens Trio	55	2.9	2.94	2300	900	1.00	1.20
Siemens Trio	6	2.9	2.94	2300	900	1.00	1.20
Siemens Trio	6	2.9	2.94	2300	900	1.00	1.20
Siemens Trio	6	3.0	2.96	2300	900	1.00	1.20
Siemens Trio	9	3.0	2.94	2300	900	1.00	1.20
Siemens Triotim	12	3.0	2.86 - 2.91	2300	900	1.00 - 1.03	1.20
Siemens Triotim	12	3.0	2.91	2300	900	1.00	1.20
Siemens Triotim	13	3.0	2.91	2300	900	1.00	1.20
Siemens Triotim	15	3.0	2.86 - 2.91	2300	900	1.00	1.20
Siemens Triotim	16	3.0	2.91	2300	900	1.00	1.20
Siemens Triotim	2	3.0	2.91	2300	900	1.00	1.20
Siemens Triotim	2	3.0	2.91	2300	900	1.00	1.20
Siemens Triotim	2	3.0	2.91	2300	900	1.00	1.20
Siemens Triotim	22	3.0	2.86 - 2.91	2300	900	1.00	1.20
Siemens Triotim	4	3.0	2.91	2300	900	1.00	1.20
